# Uncovering the first complete plastome genomics, comparative analyses, and phylogenetic dispositions of endemic medicinal plant *Ziziphus hajarensis* (Rhamnaceae)

**DOI:** 10.1186/s12864-022-08320-2

**Published:** 2022-01-27

**Authors:** Sajjad Asaf, Waqar Ahmad, Ahmed Al-Harrasi, Abdul Latif Khan

**Affiliations:** 1 Natural and Medical Sciences Research Centre, University of Nizwa, 616 Nizwa, Oman; 2 Department of Engineering Technology, University of Houston, Houston, TX 77479 USA

**Keywords:** Plastome, *Ziziphus*, SSRs, Inverted repeats, Phylogeny, Genome comparison

## Abstract

**Background:**

*Ziziphus hajarensis* is an endemic plant species well-distributed in the Western Hajar mountains of Oman. Despite its potential medicinal uses, little is known regarding its genomic architecture, phylogenetic position, or evolution. Here we sequenced and analyzed the entire chloroplast (cp) genome of *Z. hajarensis* to understand its genetic organization, structure, and phylogenomic disposition among Rhamnaceae species.

**Results:**

The results revealed the genome of Z. *hajarensis* cp comprised 162,162 bp and exhibited a typical quadripartite structure, with a large single copy (LSC) region of 895,67 bp, a small single copy (SSC) region of 19,597 bp and an inverted repeat (IR) regions of 26,499 bp. In addition, the cp genome of *Z. hajarensis* comprises 126 genes, including 82 protein-coding genes, eight rRNA genes, and 36 tRNA genes. Furthermore, the analysis revealed 208 microsatellites, 96.6% of which were mononucleotides. Similarly, a total of 140 repeats were identified, including 11 palindromic, 24 forward, 14 reverse, and 104 tandem repeats. The whole cp genome comparison of *Z. hajarensis* and nine other species from family Rhamnaceae showed an overall high degree of sequence similarity, with divergence among some intergenic spacers. Comparative phylogenetic analysis based on the complete cp genome, 66 shared genes and *mat*K gene revealed that *Z. hajarensis* shares a clade with *Z. jujuba* and that the family Rhamnaceae is the closest family to Barbeyaceae and Elaeagnaceae.

**Conclusion:**

All the genome features such as genome size, GC content, genome organization and gene order were highly conserved compared to the other related genomes. The whole cp genome of *Z. hajarensis* gives fascinating insights and valuable data that may be used to identify related species and reconstruct the phylogeny of the species.

**Supplementary Information:**

The online version contains supplementary material available at 10.1186/s12864-022-08320-2.

## Background

*Ziziphus* Mill. is one of the medicinally and economically important genera of Family Rhamnaceae. It is disseminated across the sub-tropical and warm-climatic zones of the world. The members of the *Ziziphus* genus are shrubs (with thorns) and small trees, which vary in numbers as claimed by various reports, like 86 [1, 2], 135 [3] and 170 [4]. The plant list (2013) reports 58 accepted species [5]. Two members *Z. mauritiana Lam* (ber, Indian jujube) and *Z. jujuba Mill* (common jujube), are broadly domesticated and grown commercially across the globe. Ziziphus plants hold many medicinally important phytochemicals like phenols, flavonoids, alkaloids, saponins etc., to which may be attributed their medicinal importance and pharmacological activities [6].. Traditionally, the plant cultivars were differentiated based on morphology and pedigree-related information. However, the morphology of a plant is easily affected by environmental fluctuations and hence limits the approach [7]. Furthermore, the Rhamnaceae family has more than 900 species. The available genomic sequences are only of few member plants [8]; because of the diversity in views and limited genomic information available, the intra-generic classification is a difficult problem [9]. The history of the taxonomic relationships of Rhamnaceae was reported by various researchers [10–12]. Analyses using the *rbc*L gene [13] revealed that Elaeagnaceae and Rhamnaceae had a close relationship. Similarly, the relationship between Rhamnaceae and Elaeagnaceae has been established by studies using 18S nuclear ribosomal DNA, *atpB*, and *rbcL* sequence data [14, 15].

*Z. hajarensis* is new and endemic species to Oman. It can be found in Western Hajar (Jabel al Akhdar), which shares the habitat with *Juniperux excela* subsp. *polycarpos*, while in Eastern Hajar it can be found in open woodland with *Caratonia oreothauma* subsp. *oreothauma* and *Prunus arabica. Z. hajarensis* is a multi-stemmed shrub or tree with straight, equal-length, uniform spines, juvenile branches with a prominent zig-zag pattern, and dark green leaves with entire margins [16, 17]. Locals consume the fruit and kernels directly from the tree (the fruits persist for several months on the trees). Branch bark is peeled off and used like axes to lower and cut foliage for goat fodder. The identification of this species was performed by molecular studies using chloroplast DNA microsatellites [16, 17]. The identification of *Z. hajarensis* is in confusion with *Z. spina-christi* because they share similar morphological characteristics and that is why the taxonomy of *Z. hajarensis* remains indistinct [16, 17]. Furthermore, the phylogenetic relationships of *Ziziphus* genus have been controversial and the published data on taxonomic revision is limited. Likewise, little information is available on their genetic structure, especially their chloroplast genomes or precise phylogenetic placement.

The chloroplast is one of the critical cellular organelles in plants, and serves as a reaction site for many vital biochemical reactions. It participates in the biosynthesis of starch, lipid amino acids and pigments [18]. The chloroplast has its own genome, also known as the plastome. The normal chloroplast genome of an angiosperm is a double-helix-circular DNA molecule [19]. The chloroplast genome is quite conserved in gene content, number and organization. The chloroplast genome size may vary from plant to plant, varying from 120 to 160 kb. The chloroplast genome is divided into four parts, a large-single copy of 80 to 90 kb and a small-single copy of 16 to 27 kb, set apart by two inverted repeats of 20–28 kb [20]. It has 110 to 130 unique genes that codes for photosynthesis attributed proteins, transfer RNA and ribosomal RNA [21]. The variation in chloroplast genome size is because of the genes expansion and contraction and the loss of inverted repeats. These characteristics are vital in phylogenetic and evolutionary studies [8].

The significance of the chloroplast genome, such as its organization and its role in the evolution and phylogenetic studies, has recently gained attention. Thousands of cp genomes have been sequenced and reported in National Centre for Biotechnology Information (NCBI) database. The chloroplast genome is inherited maternally, and this uniparental inheritance is proved to be very convenient in deducing the evolutionary background, phylogeographic and phylogenetic studies of plants [8]. Next-generation sequencing (NGS) is cost-friendly, time-efficient, and high throughput, enabling the chloroplast genomes to be sequenced entirely. In the *Ziziphus* genus, five chloroplast genomes have been sequenced, including *Z. jujuba, Z. acidojujuba, Z. incurva, Z. mauritiana,* and *Z. spina-christi* [9]*.* To the best of our knowledge, we report the complete chloroplast genome of *Z. hajarensis* for the first time in the current study. Considering the taxonomic and phylogenetic complications for the genus Ziziphus and lack of concentrated evidence, here, we sequenced and performed a comparative analysis of the complete chloroplast genome of *Z. hajarensis* and compared it with nine related species from the family Rhamnaceae. We predicted their relationships through a comparative analysis with other *Ziziphus* species chloroplast genome sequences within phylogenetic clades. These results reshape our understanding of the evolution of the genus *Ziziphus* and their close relatives.

## Results

### General features and Organization of Chloroplast Genome

**Fig. 1:**
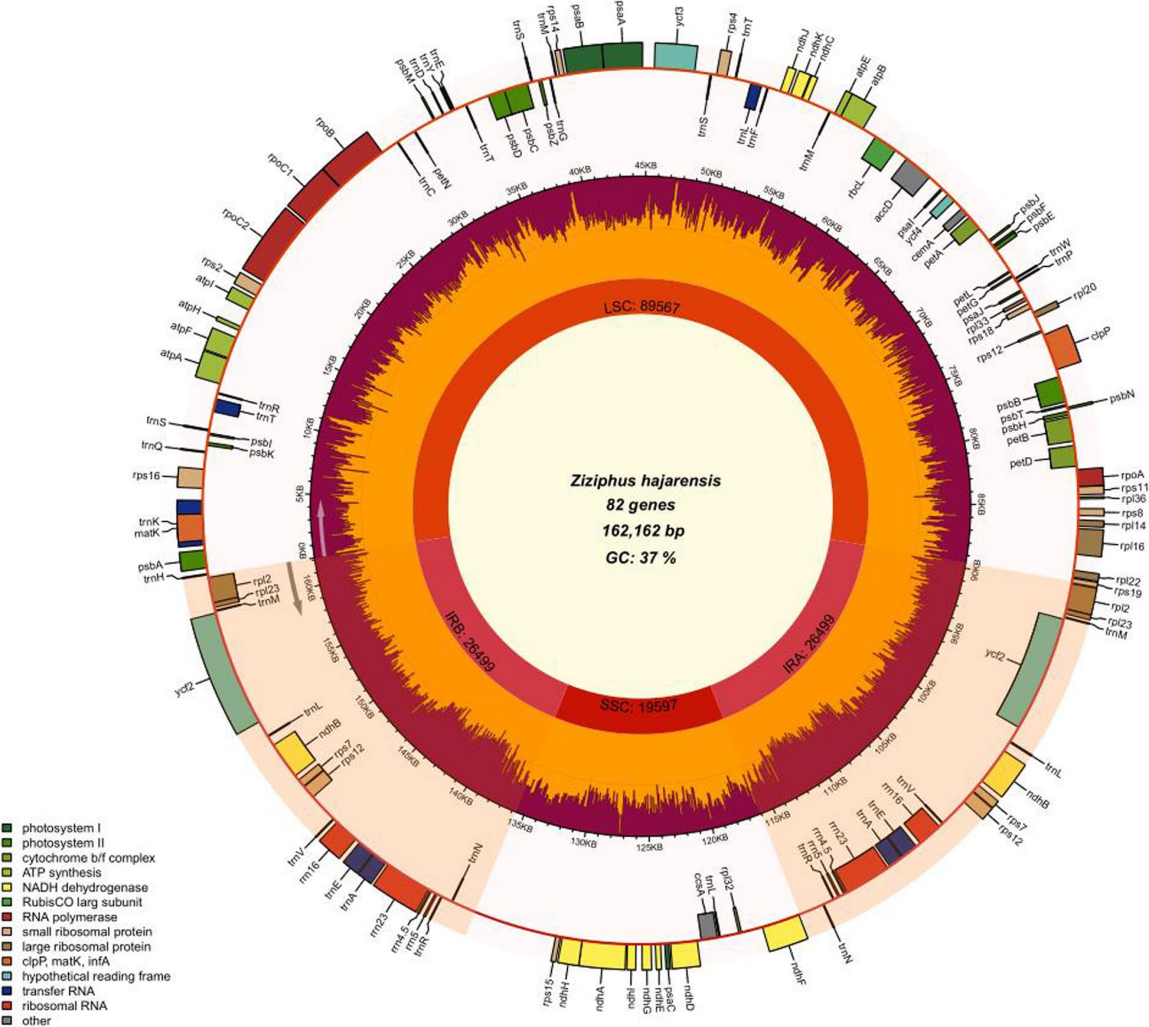
Genome Map of the *Z. hajarensis* Chloroplast genome. The thick lines represent the length of inverted repeat regions (IRs). IRs divide the chloroplast genome into Small single copy (SSC) and Large single copy (LSC) regions. Genes drawn outside the circle are transcribed counter clockwise, while those inside the circle are transcribed clockwise. Functional groups of genes are color-coded. The dark grey in the inner circle corresponds to the GC content, while the light grey corresponds to the AT content

The chloroplast genome of the *Z. hajarensis* was 162,162 bp: among the largest found among the analyzed genomes (four from *Ziziphus* genus and five from sub-family members of Rhamnaceae). These genomes thus range in size from 154,962 bp (*B. lineata*) to 162,162 bp (*Z. hajarensis*) (Fig. 1, Table 1). The cp genome of *Z. hajarensis* is a typical circular molecule organized in 4 parts (quadripartite Structure), the two IR of 26,499 bp in size, contributing 16.34% to the genome size, that divides the rest of the genome sequences into a small single copy (SSC) of 19,597 bp and large single copy (LSC) of 89,567 bp, contributing 12.08 and 55.23% respectively. The total number of encoded genes present in the chloroplast genome of *Z. hajarensis* is 126, including 82 protein-coding, eight rRNA, and 36 tRNA genes (Fig. 1, Table 1). Among the protein-coding genes, 11 genes code for ribosomal proteins of small sub-unit *(rps*2, 4, 7, 8, 11, 12, 14, 15, 16, 18, and 19) eight genes code for large subunit proteins (*rpl*14, 16, 2, 20, 23, 32, 33, and 36), five genes codes for the components of photosystem I (*psa*A, B, C, I and J), 13 genes for photosystem II (*psb*A, B, C, D, E, F, I, J, K, M, N, Z, and *ycf*3), and five genes codes for ATP synthase proteins (*atp*A, B, E, F, H, and I; Table S1). Among these annotated genes there are 17 genes (*trn*K*-UUU, rps*16*, trn*T*-CGU, atp*F*, rpoC*1*, trn*L*-UAA, trn*C*-ACA, acc*D*, rpl*2*, ndh*B*, trn*E*-UUC, trn*A*-UGC, ndh*A*, trn*A*-UGC, trn*E*-UUC, ndh*B and *rpl*2) contains a single intron while 2 genes (*ycf*3 and *clp*P) had 2 introns (Table 2). In the chloroplast genome of *Z. hajarensis* the protein-coding region is 72,917 bp in size and contributes 44.96%. Furthermore, tRNA and rRNA regions’ size was 2723 bp and 9048 bp and contributed to 1.67 and 5.57%, respectively. The overall GC content in the chloroplast genome of *Z. hajarensis* was found to be similar to other *Ziziphus* species (36.2%). In the chloroplast genome of *Z. hajarensis,* the most commonly occurring codon was ATT (*n* = 1701) encodes isoleucine followed by TTT (*n* = 1673) phenylalanine (Table S2). Contrastingly, the least common codon was TGA (*n* = 112).

**Table 1:** Summary of genome features of complete chloroplast of *Z. hajarensis* and related species from Rhamnaceae family

	*Z. hajarensis*	*Z. incurva*	*Z. jujuba*	*Z. mauritiana*	*Z. spina-christi*	*B. berchemiifolia*	*B. lineata*	*B. wilsonii*	*H. dulcis*	*R. taquetii*
Size (bp)	162,162	160,920	161,466	161,543	161,615	160,410	154,962	160,076	162,962	161,205
Overall GC contents	36.8	36.8	36.8	36.8	36.8	37.2	37	37.2	36.6	37.1
LSC size in bp	89,567	88,778	89,120	89,081	89,161	88,627	82,928	88,331	90,900	89,373
SSC size in bp	19,597	19,172	19,348	19,346	19,338	18,763	17,376	18,881	18,920	18,936
IR size in bp	26,499	26,488	26,499	26,558	26,558	26,514	27,329	26,514	26,571	26,448
Protein coding regions size in bp	72,917	79,266	73,782	78,714	78,732	72,822	78,873	79,233	80,511	78,858
tRNA size in bp	2723	2790	2722	2812	2812	2804	2796	2698	2790	2805
rRNA size in bp	9048	9044	9048	9048	9048	9048	9048	9394	9044	9048
Number of genes	126	129	133	130	130	129	131	131	130	129
Number of protein coding genes	82	84	85	84	84	82	84	83	85	84
Number of rRNA	8	8	8	8	8	8	8	8	8	8
Number of tRNAs	36	37	36	37	37	37	37	36	37	37
Genes with introns	16 + 7	15 + 8	15 + 7	15 + 8	15 + 8	15 + 8	14 + 9	16 + 6	15 + 8	15 + 8

**Table 2:** The lengths of introns and exons for the splitting genes in chloroplast genome of *Z. hajarensis*

Gene	Strand	Start	End	ExonI	IntronI	ExonII	IntronII	ExonIII
*trn*K-UUU	–	1777	4398	37	2550	35		
*rps*16	–	5113	6242	40	879	211		
*trn*T-CGU	+	9683	10,452	35	692	43		
*atp*F	–	12,968	14,252	145	748	392		
*rpo*C1	–	22,262	25,081	432	777	1611		
*ycf*3	–	45,409	47,790	124	994	230	881	153
*trn*L-UAA	+	51,183	51,832	35	565	50		
*trn*C-ACA	–	55,260	55,931	39	577	56		
*acc*D	+	61,461	63,194	633	204	897		
*clp*P	–	74,768	76,942	71	895	294	683	232
*rpl*2	–	89,724	91,233	391	685	434		
*ndh*B	–	100,408	102,620	775	680	758		
*trn*E-UUC	+	108,088	109,106	32	947	40		
*trn*A-UGC	+	109,171	110,045	37	802	36		
*ndh*A	–	126,530	129,127	805	1254	539		
*trn*A-UGC	–	141,685	142,559	37	802	36		
*trn*E-UUC	–	142,624	143,642	32	947	40		
*ndh*B	+	149,110	151,322	775	680	758		
*rpl*2	+	160,497	162,006	391	685	434		

### Genome insight, repeats and SSR analysis

**Fig. 2:**
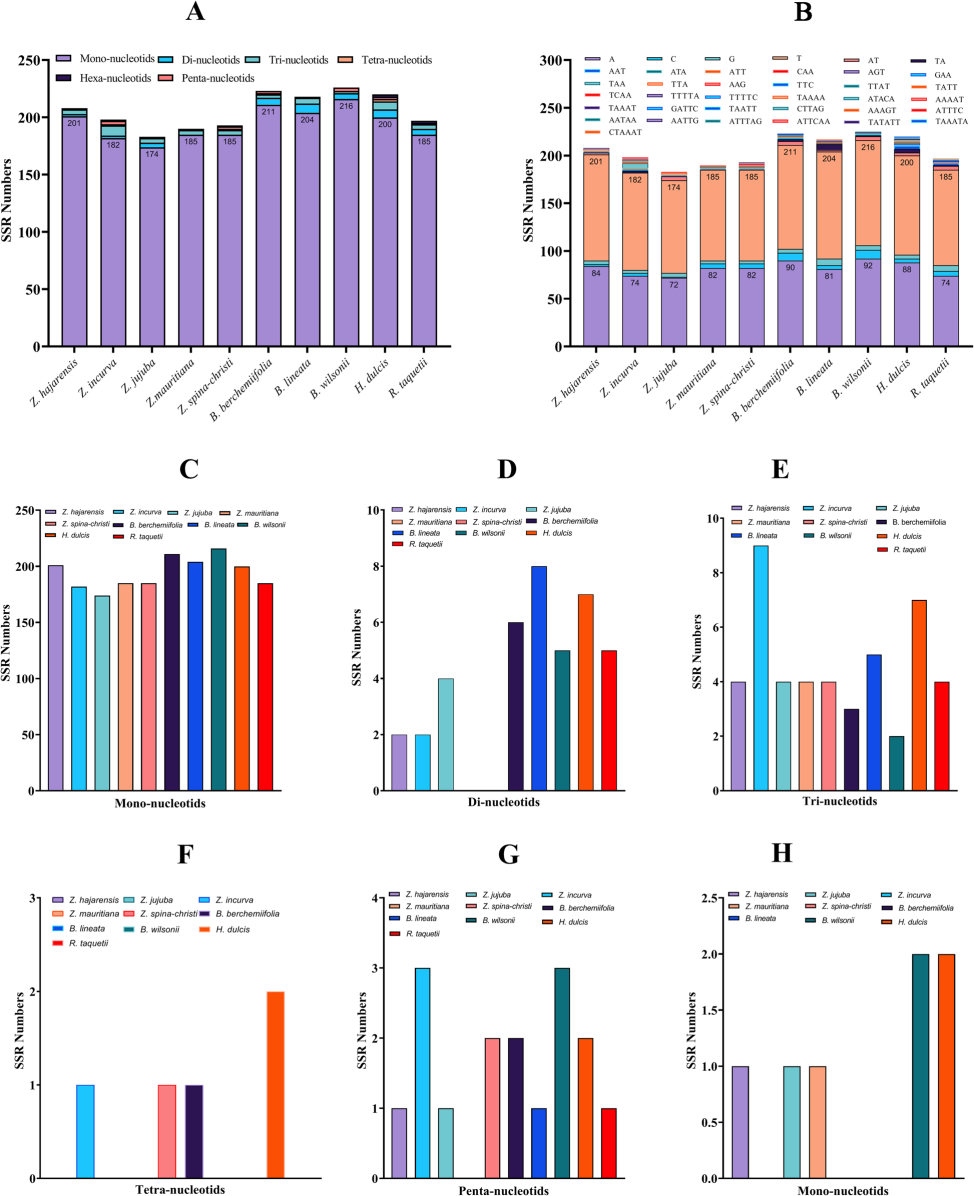
Analysis of the simple sequence repeats (SSRs) in the chloroplast genome of *Z. hajarensis* and compared cp genomes of related species; **A** Total number of SSR repeats in genomes; **B** Frequency of the simple sequence repeat motif in the chloroplast genome of *Z. hajarensis* and compared cp genomes of related species; **C** Mono-nucleotides SSRs; **D** Di-nucleotides SSRs; **E** Tri-nucleotides SSRs; **F** Tetra-nucleotides SSRs; **G** Penta-nucleotides SSRs (**H**) Hexa-nucleotides SSRs

Simple-sequence repeats (SSR) works as genetic markers in evolutionary studies and population genetics. SSR or microsatellites are sequences of 1–6 bp repeats. In this study, SSR analysis was performed for the *Z. hajarensis* chloroplast genome and nine other species from Rhamnaceae. The total identified SSR markers for each species falls between 190 to 223, including mono to hexanucleotides. In Z. *hajarensis* a total of 208 SSRs were identified, and majority are mono-nucleotides (96.6%), with two di-, four tri-, and one pentanucleotide repeat. Furthermore, the highest and lowest number of SSRs were identified in *B. wilsonii* (226) and *Z. jujuba* (183) with 94.6 and 97.3% of mono-nucleotides, respectively (Fig. 2). By exploring all four parts of *Z. hajarensis* chloroplast genome along with the coding and non-coding regions, the SSRs specified to each part and region have been identified. In the SSC and LSC regions, a total of 36 and 154 repeats have been detected, respectively. It is noteworthy that only one pentanucleotide repeat TTTTC was identified in *Z. hajarensis* specifically (Fig. 2). Furthermore, nine mono-nucleotide SSRs have been identified in IR regions of *Z. hajarensis*. Contrastingly, 30 mono-nucleotide SSRs were identified in the protein-coding region. Despite this, most SSRs were identified in intergenic regions in the genome. In the inter-genic spaces of the *Z. hajarensis* chloroplast genome, a total of 176 repeat sequences have been detected. Still, in other similar species, the number of repeats in inter-genic spaces ranges from 154 to 196 (Fig. S1).

**Fig. 3:**
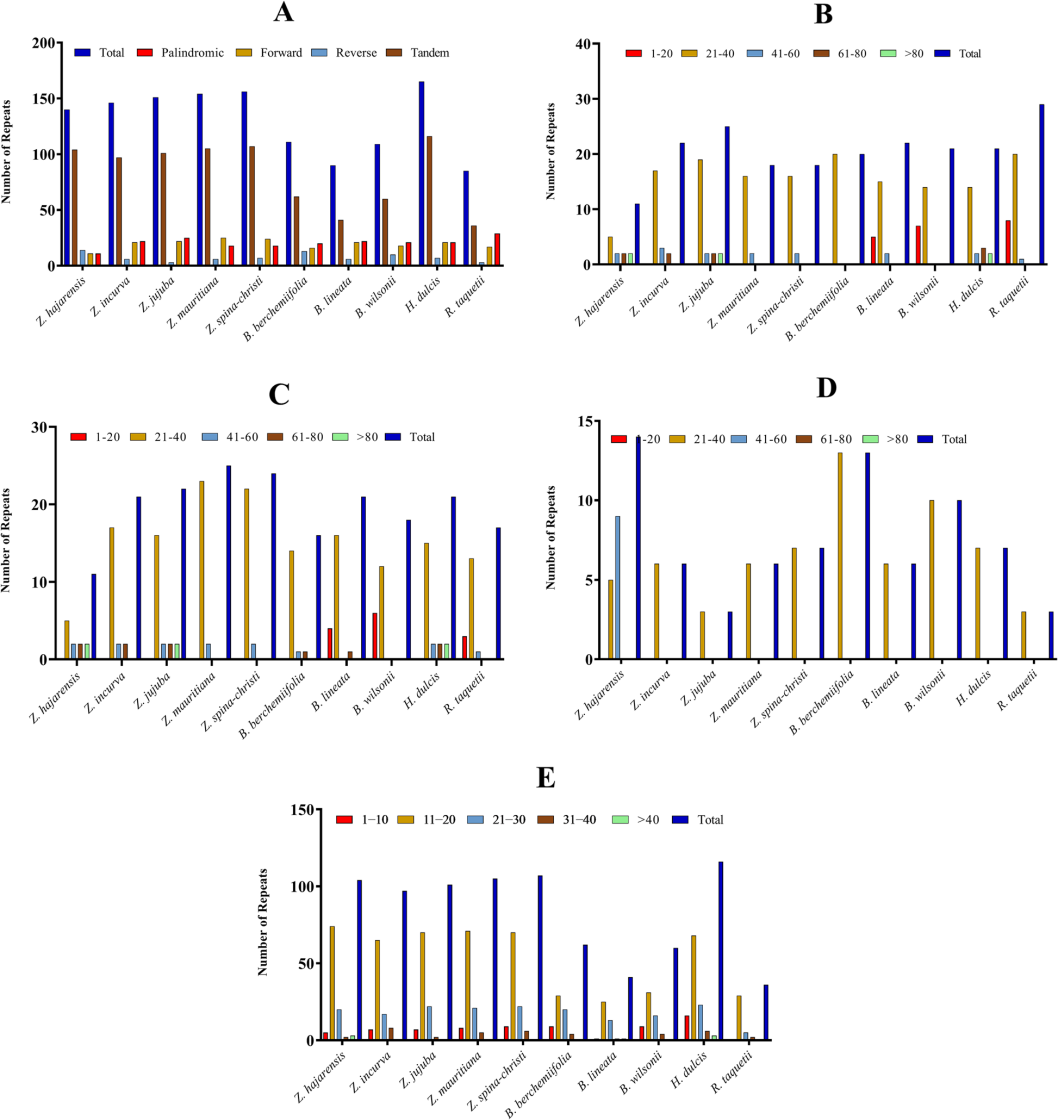
Analysis of the repetitive sequences in chloroplast genome of *Z. hajarensis* and related species. **A** A total number of repetitive sequences in cp genomes; **B** Lengthwise frequency of palindromic repeats (**C**) Lengthwise frequency of forward repeats (**D**) Lengthwise frequency of reverse repeats (**E**) Lengthwise frequency of tandem repeats

A total of 140 repeats were identified in the *Z. hajarensis* cp genome, including 11 palindromic, 24 forward, 14 reverse, and 104 tandem repeat sequences. Similarly, the lowest number of palindromic repeats and the highest number of reverse repeats were noted in the *Z. hajarensis* cp genome, 11 and 14, respectively (Fig. 3). In the chloroplast genome of *Z. hajarensis,* the lengthwise distribution of palindromic, forward, reverse. Tandem repeats were analysed in which the highest number (5 repeats each) of palindromic and forward repeats were recorded in size range of 21–40 bp, whereas the most number (9) of reverse repeats were identified in 41–60 bp size range. Similarly, for all other compared nine species, the most significant number of palindromic, forward, and reverse repeat sequences were identified in size range of 21–40 bp, as shown in Fig. 3. Furthermore, the maximum number (74) of tandem repeats were identified in range of 11–20 bp in *Z. hajarensis* and, similar results were observed in related species cp genomes (Fig. 3).

### Sequence divergence analysis

**Fig. 4:**
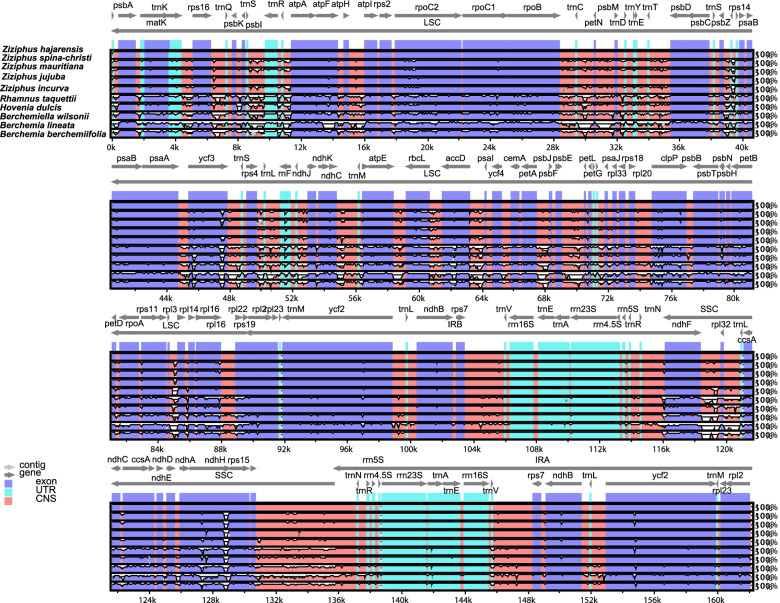
Visual alignment of chloroplast genomes of *Z. hajarensis* vs. previously reported chloroplast genomes of related species. VISTA-based identity plot showing sequence identities among 10 species, using *Z. hajarensis* as a reference. Genome regions are color-coded as protein coding, rRNA coding, tRNA coding, or conserved noncoding sequences (CNS). The x-axis represents the coordinate in the chloroplast genome. Annotated genes are displayed along the top. The sequences similarity of the aligned regions is shown as horizontal bars indicating the average percent identity between 50 and 100%

*A Z. hajarensis* cp genome comparison with related species showed sequence variation in various regions. Z. *hajarensis* was selected as reference genome. The results showed high sequence similarities among these cp genomes, especially in protein-coding and IR regions. Variations were observed in the intergenic regions such as *psb*I-*atp*A, *atp*H*-atp*I, *psb*M-*psb*D, *ycf*3-*rps*4, *ndh*C-*atp*E*, ndh*F*-rpl*32*, psb*F*-pet*G*, rps*15*-trn*N and *rpl*32*-ccs*A. In addition to these regions, some divergence was also observed in protein-coding genes such as *mat*K*, atp*F*, rpo*C2*, ycf*3*, rbc*L*, clp*P*, pet*B*, ndh*H*, ycf*2, and *psa*B (Fig. 4). The average pairwise sequence divergence was determined among the *Z. hajarensis* and other related chloroplast genomes (Table S3). The highest divergence 0.134 was found with *B. lineata,* while the lowest was found with *Z. jujuba* (0.0040). Moreover, among all the compared genes, the most divergent genes were *atp*F, *ccs*A, *clp*P, *mat*K, *ndh*F, *ndh*H, *pet*N, *rbc*L, *rpl*36 and *rpoC*2. The highest average pairwise divergence 1.408 was recorded for *rbc*L gene and followed by *pet*N gene (0.427). Furthermore, the highest divergence 1.022 was observed among the Ziziphus species in *rbc*L genes. However, in *Berchemia* species, this *rbc*L gene showed the highest divergence (4.3) among all analyzed species cp genomes. (Table S4).

### Expansion and contraction of IR regions

**Fig. 5:**
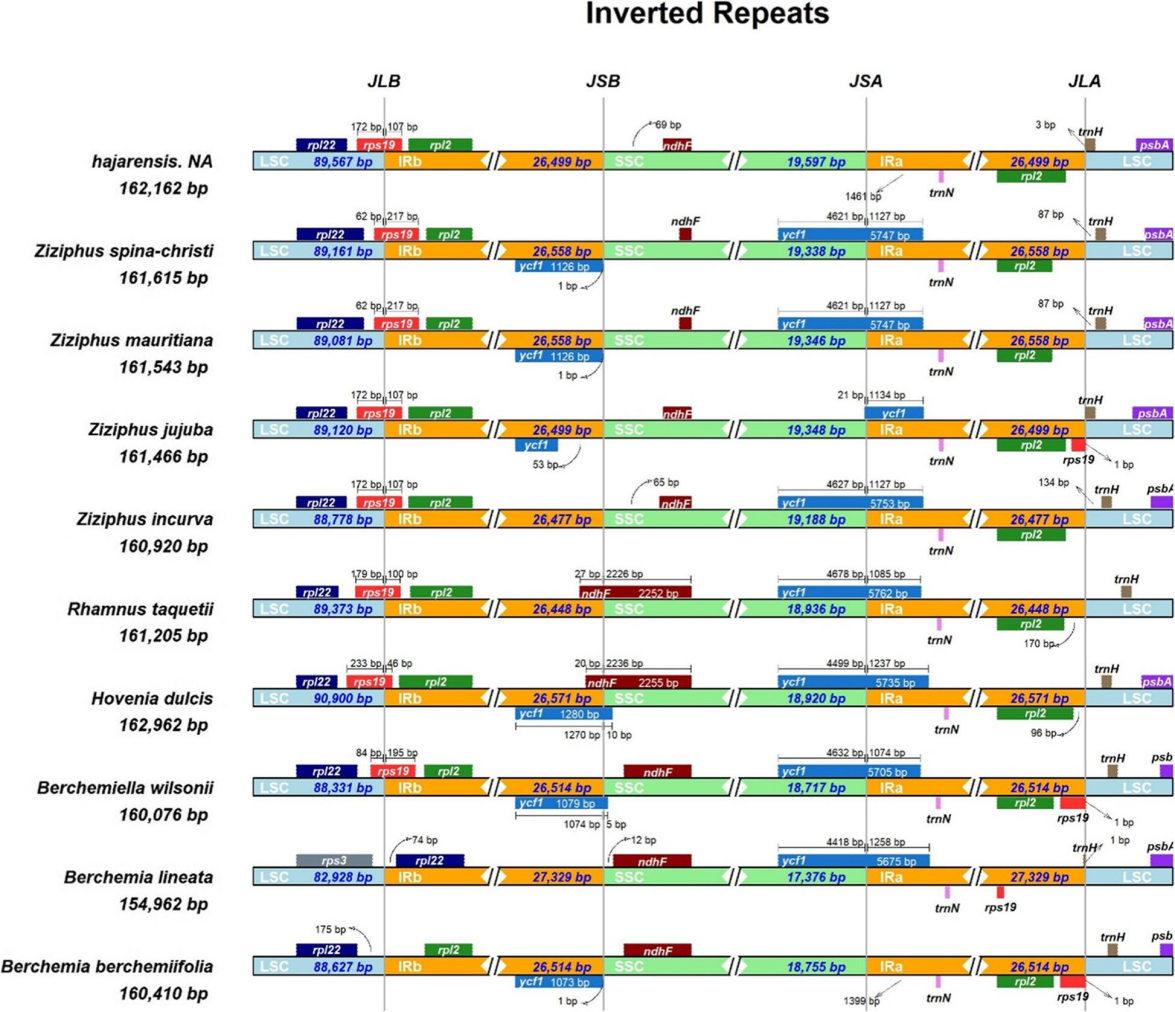
Distances between adjacent genes and junctions of the small single-copy (SSC), large single-copy (LSC), and two inverted repeat (IR) regions among chloroplast genomes of *Z. hajarensis* and related species within the Rhamnaceae family. Boxes above and below the primary line indicate the adjacent border genes. The figure is not to scale regarding sequence length and only shows relative changes at or near the IR/SC borders

In angiosperms, the variations in chloroplast genome size result from the expansion or contraction of the IR/LSC or IR/SSC regions. In this study, an extensive evaluation and comparison of all the four IRa/LSC (J^LA^), IRb/LSC (J^LB^), IRa/SSC (J^SA^) and IRb/SSC (J^SB^) junctions of the chloroplast genomes of *Z. hajarensis*, with related species cp genomes were performed. The *rps*19 gene is located at J^LB^ i.e., 172 bp in LSC and 107 bp in IRb (Fig. 5). This gene is also present at the exact location in *Z. jujuba* and *Z. incurva,* while a slight variation in *Z. spina-christi* and *Z. mauritiana,* 62 bp in LSC and 217 bp in IRb at J^LB^ in both species. The *ndh*F gene is located 69 bp away from the J^SB^ in SSC region. The *trn*N gene is located at 1461 bp from J^SA^ in the IRa region.

Furthermore, the *trn*H gene is located 3 bp away from J^LA^ in the LSC region compared to *Z. spina-christi* and *Z. mauritiana,* which is 87 bp away from J^LA^ in the LSC region. Like IR length variation was observed in genes present on IR borders with *B. lineata* cp genome. Similarly, the *rpl*22 gene is present in IRb region about 74 bp away from J^LB^ junction. Similarly, the *ndh*F gene is present in SSC region about 12 bp away from J^SB^ junction.

### Phylogenetic relationships

**Fig. 6:**
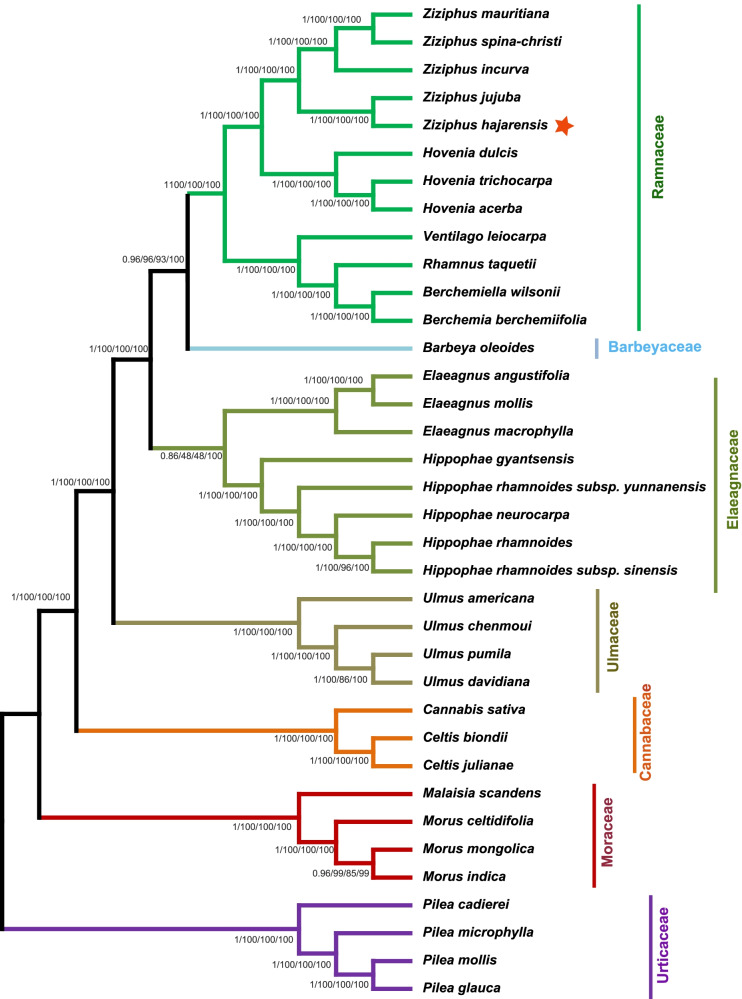
Phylogenetic trees were constructed for 36 species from seven families representing 15 genera using different methods. The entire genome dataset was analysed using four different methods: Bayesian inference (BI), maximum parsimony (MP), neighbour joining (NJ) and maximum likelihood (ML). The branches above represent bootstrap values in the ML, NJ and MP, and posterior probabilities in the BI trees. Star represents the position of Z. hajarensis

Here, the phylogenetic position of *Z. hajarensis* within the order Rosales was established by multiple alignment analysis of the complete cp genome, 66 shared protein coding genes sequences and the *mat*K gene of Rosales members representing 7 families and 15 genera (Fig. 6 and Fig. S2). Phylogenetic analysis was executed using four different methods i.e., ML (maximum likelihood), MP (maximum parsimony), NJ (Neighbour Joining) and BI (Bayesian inference). The phylogenetic trees constructed based on complete cp genomes, 66 shared genes (both nucleotides and proteins sequences) and *mat*K gene of *Z. hajarensis* formed a clade with *Z. jujuba* via bootstrap and BI support. The phylogenetic analysis revealed that *Z. hajarensis* shares the monophyletic clade with *Z. jujuba* with high bootstrap values within the phylogenetic tree based on all of the above methods. Nonetheless, the *Z. hajarensis* and *Z. jujuba* shared a sub-clade with *Z. 8ncurve*. Additionally, based on the current findings, *Ziziphus* species were in monophyletic clade with *Hovenia* species, i.e. *H. dulcis*, *H. trichocarpa* and *H. acerba*. In the analyzed data sets, Barbeyaceae and Elaeagnaceae were found the nearest families with Rhamnaceae based on the complete cp genome, 66 shared genes and *mat*K gene (Fig. 6 and Fig. S2).

## Discussion

In this study, the chloroplast genome of *Z. hajarensis* was sequenced using Ion Torrent S5 sequencing methods and compared with the available chloroplast genomes from family Rhamnaceae. The *Z. hajarensis* cp genome shared a typical structure (quadripartite) arranged circularly, with one large and one small single copy (LSC, SSC) as well as two inverted repeats (IR) regions as reported previously in angiosperms [22, 23]. The cp genomes studied here were highly conserved as reported in other angiosperms [24]. The size of *Z. hajarensis* cp genome is in agreement with the already sequenced chloroplast genomes of *Z. jujuba* (160,920 bp) and other subfamily members [8]. Similarly, the IR size of *Z. hajarensis* is 26,499 bp which falls in the typical angiosperm size range (20–28 kb) of chloroplast genomes [25]. Considering the Similar sizes of IR, it is proposed that the contributing factor to the difference of genome sizes could be the variations in LSC region as evident by [23, 26]. Furthermore, in the chloroplast genome of *Z. hajarensis,* 19 genes were identified with introns (11 protein-coding genes and 8 tRNA genes). Similar to other angiosperm cp genomes, in *Z. hajarensis* these protein coding genes have two genes (*ycf*3 and *clp*P) with two introns (Table 2).

Sequences of repetitive nature have a crucial role in rearranging and providing stability to the chloroplast genome sequences and determines the variation in copy number in different and same species. Because of the variable copy number and variation in length, the SSRs have gained considerable importance in various studies like biogeographic and population genetics [27–29]. A total of 140 repeats were identified in the chloroplast genome of *Z. hajarensis* compared to 146, 151 and 154 repeats in *Z. incurve*, *Z. jujuba* and *Z. mauritiana*. These repeats in cp genomes play a pivotal role in genomic expansions or rearrangements and structural variation and stability [30–32]. In all the compared chloroplast genomes the highest and lowest number of repeat sequences were found in *H. dulcis* (165) and *R. taquetii* (85), respectively (Fig. 2). The number of forward and reverse sequences repeats found in *Z. hajarensis* was 11 and 14 respectively. Similarly, the forward sequence repeats were the lowest and reverse sequence repeats were the highest for *Z. hajarensis* compared to the other related species. The highest number of forward repeats found were noted in *Z. mauritiana* (25), while the lowest reverse repeats were found in *Z. jujuba* and *R. taquetii* (3). Similarly, the number of palindromic sequences found in *Z. hajarensis* were 11. The number of tandem repeat sequences recorded for *Z. hajarensis* was 104. The highest value of tandem repeats was noted for *H. dulcis* (116), followed by *Z. spina-christi* (107) and *Z. jujuba* (105). In contrast, the least tandem repeats were recorded for *R. tanquetii* (36). These repeats exhibit a similar pattern as reported previously [8, 9, 33, 34]. The complex repeats and high numbers are key components in studying the chloroplast genome evolution [35, 36].

SSRs (simple sequence repeats) hold several essential characteristics, including haploid nature, relative recombination, and maternal inheritance. Because of these features, SSRs are considered one of the valuable markers. They have been diversely employed in various studies like estimating genetic variation, gene flow analysis, and exploring animal and plant populations [37–39]. The significance and applicability of SSRs markers have been reported in various other *Ziziphus* species [9, 40, 41]. In the current study, the distribution and type of SSRs markers have been analysed in *Z. hajarensis* and related cp genomes. The number of SSRs identified in *Z. hajarensis, Z. incurve*, *Z. jujuba* and *Z. mauritiana* were 208, 98, 183, and 190, respectively. Mono-nucleotide chloroplast genome SSRs in a single copy region is responsible for the intra-species chloroplast genome variations [37]. The current study’s findings agree with previously reported findings. The SSRs in chloroplast genomes combine polyadenine or polythymine repeats containing tandem guanine or cytosine repeats [42], resulting in AT-rich chloroplast genomes [43, 44].

Like typical angiosperm cp genomes, the *Z. hajarensis* shared a high sequence with all analyzed species. However, some regions showed lower sequence similarity in these cp genomes. As reported previously, the sequence divergence recorded in the IR region was lower than LSC and SSC regions [25]. These results agree with previous reports that showed a higher sequence divergence because of copy correction for gene conservation in IR regions [45, 46]. The *Z. hajarensis* cp genome showed high sequencing divergence in various intergenic regions and genes like *psb*I-*atp*A, *atp*H*-atp*I, *psb*M-*psb*D, *ycf*3-*rps*4, *mat*K*, atp*F*, rpo*C2*, ycf*3*, rbc*L and *clp*P (Fig. 4). The average pairwise sequence divergence was calculated and *Z. hajarensis* showed an average pairwise sequence divergence of 0.041with related species cp genomes. The highest divergence was observed with *B. lineata.* Similarly,the *rbc*L gene was found with the highest pairwise sequence divergence followed by *pet*N gene. Comparable findings for these genes have previously been reported [26, 47, 48], and our results are supported by Yang et al. [49] suggesting that similar variations exist among different coding regions. These findings are also supported by earlier report that these divergent genes are primarily found in LSC regions and are evolving rapidly [47]. Extensive IRs play a significant role in maintaining the conserved structure and stability of chloroplast genome [50, 51]. Variations in length among the cp genomes were observed because of the expansion or contraction of IR regions [52, 53]. An IR copy was lost in the plastomes of tribes in the legume subfamily Papilionoideae [54] during the evolution of the angiosperms, and cp genome rearrangements are more common in these species compared with species possessing typical IRs [55]. We evaluated and compared all four IRa/LSC (J^LA^), IRb/LSC (J^LB^), IRa/SSC (J^SA^) and IRb/SSC (J^SB^) junctions of the cp genome of *Z. hajarensis*, with related species. The *rps*19 gene in *Z. hajarensis*, *Z. jujuba* and *Z. incurva* was found the same, which is 172 bp in LSC and 107 bp in IRb (J^LB^). In contrast, the same gene location is slightly different in *Z. spina-christi* and *Z. mauritiana* (Fig. 5). Similarly, variations were also noted with *B. lineata* in the location of *rpl*22 gene present on IR borders. Previous studies have revealed that there is an expansion of the IR and LSC regions in angiosperm plastomes during evolution [23, 56, 57].

Chloroplast genomes have been played a significant role in molecular, evolutionary and phylogenetic studies. Analyses based on complete chloroplast genome sequence comparison have solved numerous phylogenetic problems at the deep node level. They have contributed to understanding less known evolutionary associations between angiosperms [26, 58]. Several phylogenetic studies have been conducted on the subfamily Rhmnaceae and intra-generic within *Ziziphus* but could not address the problems related to the classification of *Z. hajarensis* based on ITS regions, SSRs regions, *rbc*L gene etc. [59–61]. On the other hand, complete genome sequencing provides more in-depth information [43, 45, 62]. The complete cp genome sequence of *Z. hajarensis* has been overlooked in this respect; the new dataset will give more detailed insights into the role of different genes, allowing for a better knowledge of the plant’s history. Chloroplast genomes have proven to be helpful in phylogenetic analyses and molecular and evolutionary systematics. In recent years, various studies based on the entire cp genome and compared with many protein-coding genes have been undertaken at deep nodes to solve phylogenetic problems [63, 64]. This approach allows a better understanding of the complex evolutionary links among angiosperms [58]. Therefore, in this study, the phylogenetic position of *Z*. *hajarensis* within Rhamnaceae and Rosales was established by utilizing the complete cp genomes, 66 shared genes proteins coding genes and *mat*K gene among the members of 7 families representing 15 genera Four different methods, which are ML (maximum likelihood), MP (maximum parsimony), NJ (neighbor-joining) and BI (Bayesian inference), were used for phylogenetic analysis*.* The results revealed that complete cp sequences (Fig. 6), 66 shared genes (Fig. S2 A and *mat*K gene (Fig. S2 B) from all the analysed species generated a phylogenetic tree with the same topology. In these phylogenetic trees (Fig. 6 & S2) constructed by employing ML, MP, NJ, and BI methods, *Z. hajarensis* formed a single clade with *Z.jujuba* with high bootstrap (100%) and BI support*.*

Furthermore, Barbeyaceae and Elaeagnaceae were the nearest families to the Rhamnaceae. Similar results were reported previously based on chloroplast *rbc*L and *atp*B genes where Elaeagnaceae and Rhamnaceae showed a close relationship [13]. Similarly, phylogenetic study based on plastid non-coding region revealed family Barbeyaceae and Dirachmaceaein in a close relationship with Rhamnaceae [14, 15]. These findings also suggest that Rhamnaceae germplasm-related genetic resources are important and valuable for Rhamnaceae species identification, phylogenetic inference, and taxonomy clarification. Furthermore, if plastid genomes are made accessible, phylogenetic inferences within Rosales and Rhamnaceae might be improved, potentially offering hundreds of useful molecular markers for future studies.

## Conclusion

For the first time, the current findings provide comprehensive insights into the entire cp genome of *Z. hajarensis*. The structure and gene content of the *Z. hajarensis* cp genome was determined to be in synergy with similar Rhamnaceae species. We retrieved important genetic characteristics such as repetitive sequences, SSRs, codon use, IR contraction and expansion, sequence divergence, and phylogenomic position using thorough bioinformatic analysis and comparative assessments. Repetitive sequences like tandem repeats and SSRs were examined within these cp genomes. Overall, there was a significant sequence similarity amongst these cp genomes. However, these cp genomes had several divergent genes and intergenic regions, including *psb*I-*atp*A, *atp*H-*atp*I, *psb*M-*psb*D, *mat*K, *atp*F *rpo*C2, *ycf*3, and *rbc*L. The current work presents a valuable set of complete chloroplast genome analyses of *Z. hajarensis* and related species, which might aid in species identification and biology, genetic diversity, and phylogenetic studies.

## Methods

### Sample collection

The fresh juvenile leaves were collected from *Z. hajarensis* plant growing in natural habitat of Jabal Al-Akhdar, Oman (23° 6′ 11.110″ N; 57° 22′ 47.14″ E). The climate of natural habitat is high in temperature and low in precipitation, with an average temperature of 25–46 °C. Permission (6210/10/73) to collect plants for research purposes was obtained from Ministry of Environment & Climate Affairs, Muscat Oman. The voucher specimen (UoN-ZH1) was deposited in the Herbarium Centre, University of Nizwa as plant was identified by lead taxonomist (Saif Al-Hathmi) at Oman Botanic Garden, Muscat Oman. Collected leaves were put in zipper bags, immediately kept in liquid nitrogen, and stored at − 80 °C for DNA extraction.

### DNA extraction and sequencing

The chloroplast DNA of *Z. hajarensis* was extracted from its finely powdered leaves according to the protocol [65] with brief modifications. Genomic libraries were prepared according to the provided instructions (Life Technologies USA, Eugene, OR, USA). Ion Shear™ Plus Reagents kit and Ion Xpress™ Plus, gDNA Fragment Library kit, were used to arrange the chloroplast DNA into the 400 bp fragments enzymatically and construct libraries. The quantification of prepared libraries was performed by Qubit 3.0 fluorometer and bioanalyzer (Agilent 2100 Bioanalyzer system, Life Technologies USA) and followed by the amplification of template using Ion OneTouch™ 2. By Ion OneTouch™ ES enrichment system, the templates (amplified) were enriched using Ion 530 & 520 OT2 Reagents. For sequencing, Ion sample loading on S5 530 Chip was performed according to the Ion S5 protocol.

### Genome assembly and annotation

A total of 1,315,423 raw reads were obtained for *Z. hajarensis* cp genome*.* The *Z. jujuba* was used as a reference genome for mapping the produced reads by Bowtie2 (v.2.2.3) [66] in Geneious Pro (v.10.2.3) software [67]. Assembly means coverage of *Z. hajarensis*. The annotation of the chloroplast genome of *Z. hajarensis* was performed by using CpGAVAS [68] and DOGMA [69] (http://dogma.ccbb.utexas.edu/, China). TRNAs can-SE (v.1.21) [70] software was utilized to detect tRNA genes. Furthermore, Geneious Pro (v.10.2.3) [67] and tRNAs can-SE (v.1.21) [70] were used for manual alteration and comparison of the genome (*Z. hajarensis*) with previously published *Z. jujuba* genome and intron boundaries. These tools were also utilized to adjust the start and stop codons manually. Illustration of chloroplast genome OGDRAW [71] was used for structural features. Moreover, genome divergence was determined by mVISTA [72] in shuffle-LAGAN mode, while *Z. hajarensis* chloroplast genome was chosen as the reference genome.

### Repeat identification

Forward reverse and palindromic repeats were identified on the REPuter online tool [73] with a minimum repeat size of eight bp and maximum computed repeats of 50. Similarly, SSRs were identified using MISA Software [74] with requirements set to ≥10 repeat units for one bp repeats; ≥8 repeat units for two bp repeats; ≥4 repeat units for 3 and 4 bp repeats, and ≥ three repeat units for 5 and 6 bp repeats. Tandem Repeats Finder v.4.09 [75] online tool was used to identify the tandem repeats.

### Genome divergence and phylogenetic analysis

The sequence divergence in the complete chloroplast genome and shared genes were determined among the *Ziziphus* and closely related species. After multiple sequence alignment, a comparative analysis strategy was used to compare gene order to identify the ambiguous and missing gene annotation. MAFFT version 7.222 [76] was used to align complete chloroplast genomes using default settings. Pairwise sequence divergence was determined using Kimura’s two parameter model (K2P) [76]. The phylogenetic position of *Z. hajarensis* within the order Rosales and sub-family Rhamnaceae was inferred using 36 chloroplast genome sequences retrieved from the NCBI database. First the complete chloroplast genomes were aligned based on chloroplast genome structures and conserved gene orders [77]. The phylogenetic tree was inferred on four different methods: Bayesian inference (BI), implemented in Mr. Bayes 3.1.2 [78], maximum parsimony (MP) using PAUP 4.0 [79], and maximum likelihood (ML) using MEGA 6 [80], using already described settings [25, 45]. The GTR + G substitution model was tested via jModel Test version v2.1.02 [81] as per AIC (Akaike information criterion) for Bayesian posterior probabilities in BI analysis. The Markov Chain Monte Carlo (MCMC) method was run using four incrementally heated chains across 1,000,000 generations, starting from random trees and sampling 1 out of every 100 generations. The first 30% values were considered as burn-in and discarded. In Maximum Parsimony run heuristic search was used with random addition of 1000 sequence replicates with the tree-bisection-reconnection (TBR) branch-swapping tree search criterion to estimate the posterior probabilities.

Furthermore, for ML run, the parameters were optimized by BIONJ tree [82] as the starting tree with 1000 bootstrap replicates by employing the Kimura 2-parameter model with invariant sites gamma-distributed rate heterogeneity. A set of 66 shared genes and *mat*K gene from the plastome genomes of 36 Rosales species were aligned using MAFFT version 7.22294 under default parameters and made various manual adjustments to preserve and improve reading frames in the second and third tiers of phylogenetic analysis. The above four phylogenetic inference models (ML, MP, NJ, and BI) were used to construct trees utilizing 66 concatenated genes, *mat*K gene, and 66 concatenated protein sequences as reported previously [83].

## Supplementary Information

**Additional file 1: Figure S1.** Analysis of the simple sequence repeats (SSRs) in the chloroplast genome of *Z. hajarensis* and compared cp genomes of related species. (A) Frequency of SSRs in coding and intergenic regions (B) Frequency of SSRs in inverted repeats (IR) small single copy (SSC) and large single copy (LSC) regions. **Figure S2.** Phylogenetic trees were constructed for 36 species from seven families representing 15 genera using different methods and the tree is shown for 66 protein coding shared genes (A) and *mat*K (B) data sets. These sequences data sets were used with four different methods: Bayesian inference (BI), maximum parsimony (MP), neighbour joining (NJ) and maximum likelihood (ML). The branches above represent bootstrap values in the ML, NJ and MP, and posterior probabilities in the BI trees. **Table S1.** Gene composition in *Z. hajarensis* chloroplast genome.

**Additional file 2: Table S2.** Codon Usage in *Z. hajarensis* chloroplast genome.

**Additional file 3: Table S3.** Average pairwise distance of complete chloroplast sequence from *Z. hajarensis* with related species.

**Additional file 4: Table S4.** Average pairwise distance of chloroplast shared genes from *Z. hajarensis* with related species.

## Data Availability

All data generated or analysed during this study are included in this article. The *Ziziphus hajarensis* genome deposited to NCBI (MZ475300), https://www.ncbi.nlm.nih.gov/nuccore/MZ475300.
