# Supplementary material for: Uncovering the first complete plastome genomics, comparative analyses, and phylogenetic dispositions of endemic medicinal plant Ziziphus hajarensis (Rhamnaceae)
Source: BMC Genomics. 2022 Jan 27;23:83. doi: 10.1186/s12864-022-08320-2 (PMC8796432; doi:10.1186/s12864-022-08320-2)
Supplement: Supplementary file 1 — Additional file 1: Figure S1. Analysis of the simple sequence repeats (SSRs) in the chloroplast genome of Z. hajarensis and compared cp genomes of related species. (A) Frequency of SSRs in coding and intergenic regions (B) Frequency of SSRs in inverted repeats (IR) small single copy (SSC) and large single copy (LSC) regions. Figure S2. Phylogenetic trees were constructed for 36 species from seven families representing 15 genera using different methods and the tree is shown for 66 protein coding shared genes (A) and matK (B) data sets. These sequences data sets were used with four different methods: Bayesian inference (BI), maximum parsimony (MP), neighbour joining (NJ) and maximum likelihood (ML). The branches above represent bootstrap values in the ML, NJ and MP, and posterior probabilities in the BI trees. Table S1. Gene composition in Z. hajarensis chloroplast genome. [file 12864_2022_8320_MOESM1_ESM.docx]

**Supplementary information.**


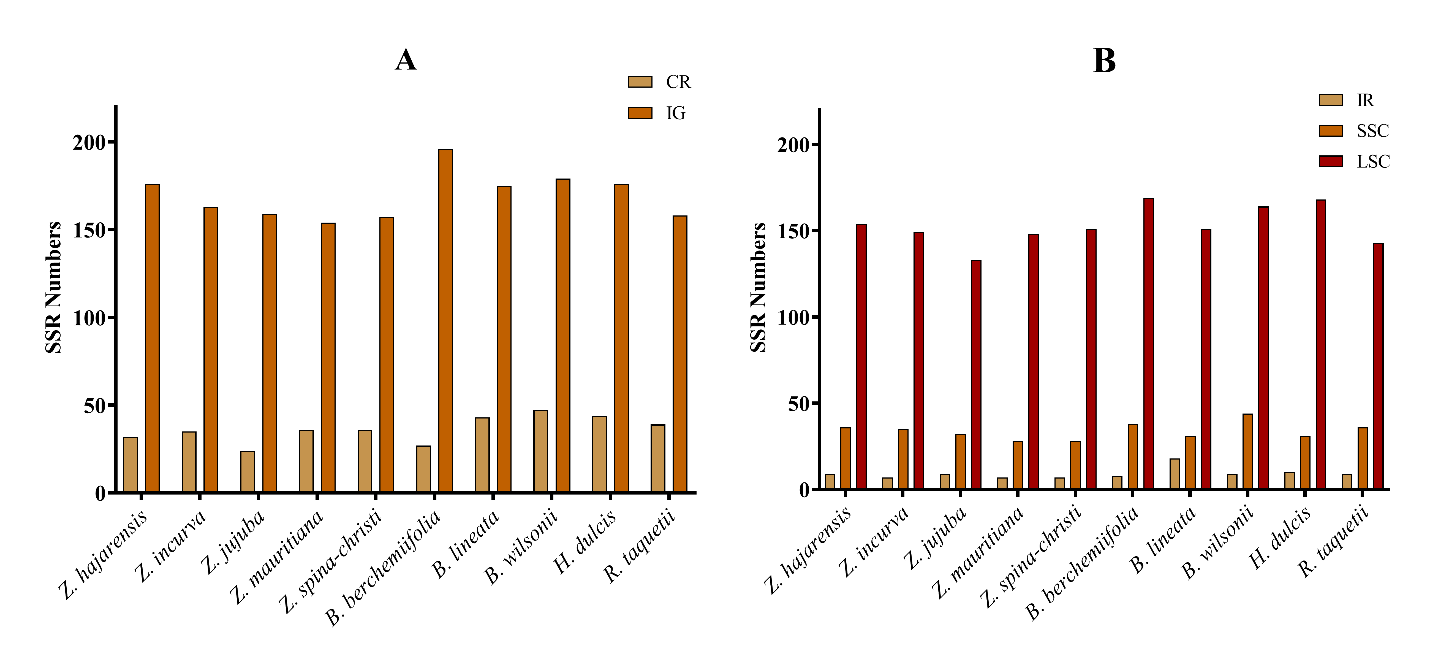


**Figure S1.** Analysis of the simple sequence repeats (SSRs) in the chloroplast genome of *Z. hajarensis* and compared cp genomes of related species. (A) Frequency of SSRs in coding and intergenic regions (B) Frequency of SSRs in inverted repeats (IR) small single copy (SSC) and large single copy (LSC) regions.


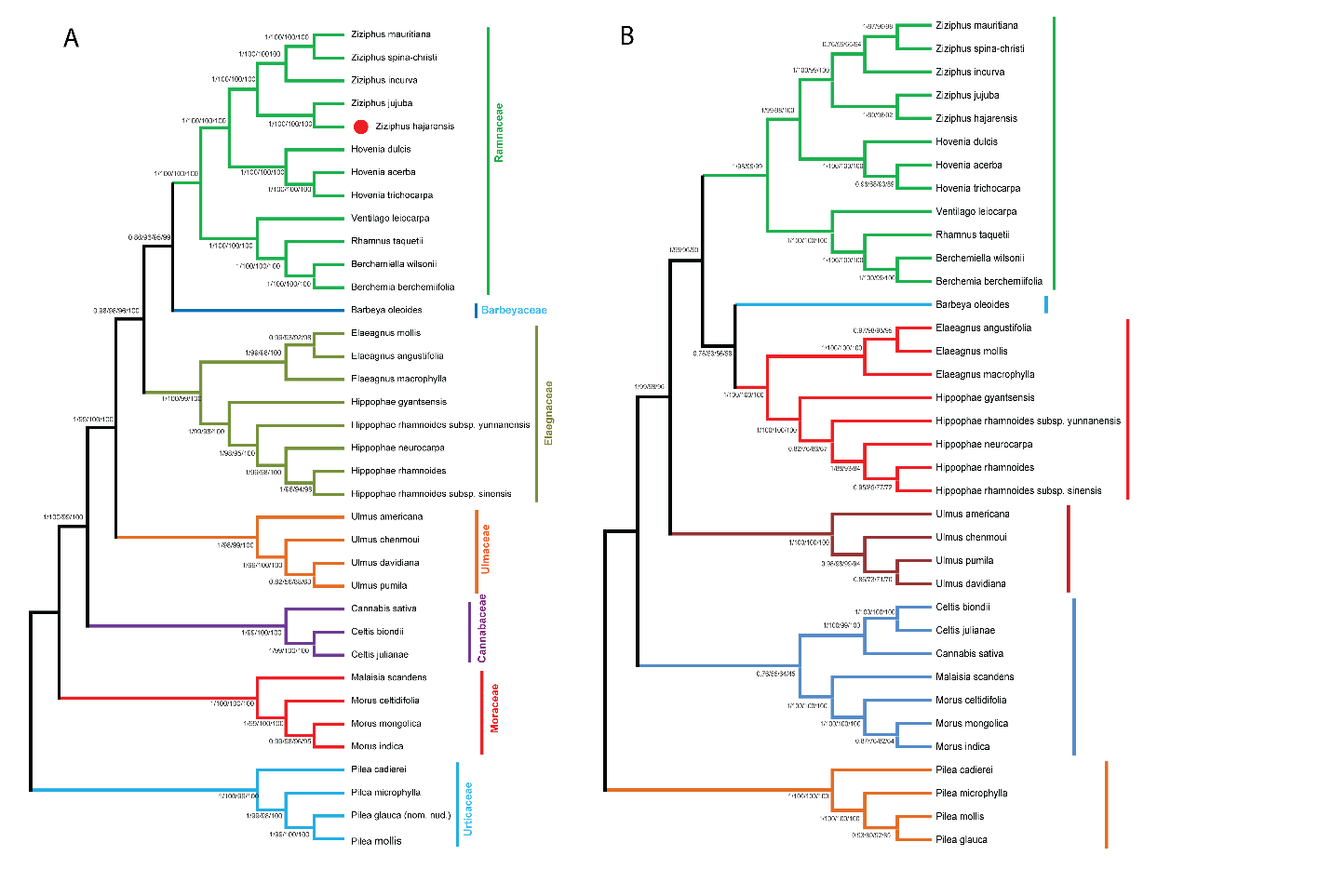


**Figure S2.** Phylogenetic trees were constructed for thirty-six species from seven families representing 15 genera using different methods and the tree is shown for 66 protein coding shared genes (A) and *mat*K (B) data sets. These sequences data sets were used with four different methods: Bayesian inference (BI), maximum parsimony (MP), neighbour joining (NJ) and maximum likelihood (ML). The branches above represent bootstrap values in the ML, NJ and MP, and posterior probabilities in the BI trees

| **Category of genes** | **Group of genes** | **Name of genes** |
| --- | --- | --- |
| Genes for photosynthesis | Subunits of ATP synthase | *atp*A, *atp*B, *atp*B, *atp*B, *atp*E, *atp*F, *atp*H, *atp*I |
|  | Subunits of photosystem II | *psb*A, *psb*B, *psb*C, *psb*D, *psb*E, *psb*F, *psb*I, *psb*J, *psb*K, *psb*M, *psb*N, *psb*Z, *ycf*3 |
|  | Subunits of NADH-dehydrogenase | *ndh*A, *ndh*B, *ndh*B, *ndh*C, *ndh*D, *ndh*E, *ndh*F, *ndh*G, *ndh*H, *ndh*I, *ndh*J, *ndh*K |
|  | Subunits of cytochrome b/f complex | *pet*A, *pet*D, *pet*G, *pet*L, *pet*N |
|  | Subunits of photosystem I | *psa*A, *psa*B, *psa*C, *psa*I, *psa*J |
|  | Subunit of rubisco | *rbc*L |
| Self-replication | Large subunit of ribosome | *rpl*14, *rpl*16, *rpl*2, rpl2, *rpl*20, *rpl*23, *rpl*23, *rpl*32, *rpl*33, *rpl*36 |
|  | DNA dependent RNA polymerase | *rpo*A, *rpo*B, *rpo*C1, IC2 |
|  | Small subunit of ribosome | *rps*11, *rps*12, *rps*12, *rps*14, *rps*15, *rps*16, *rps*18, *rps*19, *rps*2, *rps*4, *rps*7, *rps*7, *rps*8 |
| Other genes | Subunit of Acetyl-CoA-carboxylase | *acc*D |
|  | c-type cytochrom synthesis gene | *ccs*A |
|  | Envelop membrane protein | *cem*A |
|  | Protease | *clp*P |
|  | Maturase | *mat*K |
| Unknown | Conserved open reading frames | *ycf*2, *ycf*2, *ycf*4 |

Table S1. Gene composition in *Z. hajarensis* chloroplast genome.
